# Triple oral beta-lactam containing therapy for Buruli ulcer treatment shortening

**DOI:** 10.1371/journal.pntd.0007126

**Published:** 2019-01-28

**Authors:** María Pilar Arenaz-Callao, Rubén González del Río, Ainhoa Lucía Quintana, Charles J. Thompson, Alfonso Mendoza-Losana, Santiago Ramón-García

**Affiliations:** 1 Research & Development Agency of Aragon (ARAID) Foundation, Zaragoza, Spain; 2 Global Health R&D, GlaxoSmithKline, Tres Cantos, Madrid, Spain; 3 Mycobacterial Genetics Group. Department of Microbiology, Preventive Medicine and Public Health. Faculty of Medicine, University of Zaragoza, Zaragoza, Spain; 4 Department of Microbiology and Immunology, University of British Columbia, Vancouver, B.C. Canada; Kwame Nkrumah University of Science and Technology, GHANA

## Abstract

The potential use of clinically approved beta-lactams for Buruli ulcer (BU) treatment was investigated with representative classes analyzed *in vitro* for activity against *Mycobacterium ulcerans*. Beta-lactams tested were effective alone and displayed a strong synergistic profile in combination with antibiotics currently used to treat BU, i.e. rifampicin and clarithromycin; this activity was further potentiated in the presence of the beta-lactamase inhibitor clavulanate. In addition, quadruple combinations of rifampicin, clarithromycin, clavulanate and beta-lactams resulted in multiplicative reductions in their minimal inhibitory concentration (MIC) values. The MIC of amoxicillin against a panel of clinical isolates decreased more than 200-fold within this quadruple combination. Amoxicillin/clavulanate formulations are readily available with clinical pedigree, low toxicity, and orally and pediatric available; thus, supporting its potential inclusion as a new anti-BU drug in current combination therapies.

## Author summary

Buruli ulcer (BU) is a chronic debilitating disease of the skin and soft tissue, mainly affecting children and young adults in tropical regions. Before 2004, the only treatment option was surgery; a major breakthrough was the discovery that BU could be cured in most cases with a standard treatment that involved 8 weeks of combination therapy with rifampicin and streptomycin. However, the use of streptomycin is often associated with severe side effects such as ototoxicity, or nephrotoxicity. More recently, a clinical trial demonstrated equipotency of replacing the injectable streptomycin by the clarithromycin, which is orally available and associated with fewer side effects. BU treatment is now moving toward a full orally available treatment of clarithromycin-rifampicin. Although effective and mostly well tolerated, this new treatment is still associated with side effects and only moxifloxacin is additionally recommended by WHO for BU therapy. New drugs are thus needed to increase the number of available treatments, reduce side effects, and improve efficacy with treatments shorter than 8 weeks. In this work, we describe for the first time the potential inclusion of beta-lactams in BU therapy. More specifically, we propose the use of amoxicillin/clavulanate since it is oral, suitable for the treatment of children, and readily available with a long track record of clinical pedigree. Its inclusion in a triple oral therapy complementing current combinatorial rifampicin-clarithromycin treatment has the potential to counteract resistance development and to reduce length of treatment and time to cure.

## Introduction

Buruli ulcer (BU) is a chronic debilitating mycobacterial disease of the skin and soft tissue. Although mortality is low, permanent disfigurement and disability are high, mainly affecting children and young adults. BU is found primarily in tropical regions of Africa, South America and the Western Pacific; however, it is also becoming a public health concern in some regions of Australia [1].

Before 2004, when the World Health Organization (WHO) published provisional guidance for the management of BU disease [2], antibiotics were viewed as relatively ineffective and surgery remained the mainstay of treatment for BU [3–5]. In the late 1990s and early 2000s, however, *in vitro* studies demonstrated anti-BU activity of some antibiotics used for the treatment of tuberculosis (TB) and other non-tuberculous mycobacteria [6–8], and *in vivo* studies the potential for combining two drugs to provide improved treatment outcomes [9–13]. Soon after, clinical evidence showed the effectiveness of a combination of rifampicin plus streptomycin when it was administered for at least 4 weeks [14], and that routine implementation of such a therapy was possible in the field [15, 16]; however, the use of the injectable streptomycin is often associated with adverse events and it is restricted in the treatment of pregnant women and young infants. In addition, the lack of an efficacious oral treatment remained one of the main obstacles to decentralizing care at local level in rural areas. These limitations motivated the scientific community to evaluate alternative oral treatments and clinical studies demonstrated that fluoroquinolones [17] or clarithromycin [18–20] could also be used in combination with rifampicin and were associated with fewer side effects compared to the injectable streptomycin. Thus, on March 24^th^, 2017, WHO recommended full oral treatment of 8 weeks daily combination therapy of rifampicin-clarithromycin [21].

While recommended regimens (rifampicin plus streptomycin or clarithromycin) allow cure of small lesions (<5 cm in diameter) without surgery [15, 18], controversy remains regarding the best surgery approach for large lesions (>10 cm) [22, 23]. Intermittent drug administration using rifapentine, a rifampicin analog with longer half-life, instead of daily rifampicin, has been also proposed as a strategy to facilitate treatment supervision in the field [24]. However, *M*. *ulcerans* strains resistant to rifampicin have been isolated after experimental chemotherapy in mice [25] and a recent report described the emergence of *M*. *ulcerans* strains resistant to rifampicin and streptomycin in the clinic [26]; further experiments would be, however, needed to identify the genetic basis of such resistance patterns and confirm the emergence of resistance in *M*. *ulcerans* clinical isolates. Nevertheless, these reports should be a warning sign since no alternatives for rifampicin are currently available. WHO currently recommends only four drugs for the treatment of BU: rifampicin, streptomycin, clarithromycin and moxifloxacin [2]. It would be thus desirable to increase the number of drugs available to treat BU and to develop a new therapy that would reduce both duration of treatment and time to healing after therapy completion for all type of lesions and suitable for children and pregnant women.

Drug discovery and development for neglected diseases is especially delayed due to lack of interest from the main scientific and industrial communities. To speed up the process in the BU field, we applied knowledge gathered in TB R&D drug repurposing programs [27–30] where we (and others [31]) showed that beta-lactams strongly increased the bactericidal and sterilizing properties of rifampicin [28]. Rifampin is the cornerstone drug for TB (and BU) therapy with a direct relation between dose increase and therapy efficacy [32] due to its bactericidal and sterilizing activity in a dose-dependent manner [33]. However, the current WHO recommended 10 mg/kg (600 mg daily) is not its optimal clinical dosage [34] and some recent studies suggest that it could be safely increased to 35 mg/kg daily for TB therapy with a bacteriological effect on time to culture conversion [32, 35, 36]. More recently, dose-ranging high-dose rifampicin studies using a murine model of *M*. *ulcerans* disease showed that shorter BU treatments might be also feasible [37], suggesting that synergistic partners could serve to improve rifampicin efficacy without compromising tolerability and toxicity.

Beta-lactams are one of the largest groups of antibiotics available today with an exceptional record of clinical safety in humans [38]. Used for decades, they had been traditionally considered ineffective for the treatment of mycobacterial infections (mainly TB) due to the presence of a beta-lactamase (BlaC) and the hydrophobic nature of the mycobacterial cell envelope [39]. However, after a seminal publication describing the *in vitro* activity of meropenem plus clavulanate against multi-drug (MDR) and extensively drug resistant (XDR) strains of *M*. *tuberculosis* [40] and its anecdotal use in salvage therapies for XDR patients [41], the first study convincingly demonstrating the clinical efficacy of beta-lactams was recently published [29]. These studies provided evidence of their anti-mycobacterial clinical potential, opening a new avenue to identify new drugs and optimize current BU therapy.

In this study, we are translating knowledge and concepts of drug repurposing and synergy generated in TB R&D programs to assess the potential inclusion of beta-lactams for BU therapy. We propose the combination of amoxicillin/clavulanate as a new anti-BU treatment in combination with current oral BU therapy, rifampicin and clarithromycin, with the potential of treatment shortening and readily implementation in the field.

## Materials and methods

### Bacterial strains, growth conditions and reagents

*M*. *ulcerans* strain NCTC 10417 (ATCC Number: 19423; Lot Number: 63210551) was used for initial screening assays. Further validation studies were performed with clinical isolates from different geographical origins: ITM 063846, Benin; ITM 070290, China; ITM 083720 and ITM C05143, Mexico; ITM 941327, ITM C05142 and ITM M000932, Australia; ITM C05150, DR Congo; ITM C08756, Japan, purchased from the Belgian Co-ordinated Collection of Micro-organisms (BCCM).

*M*. *ulcerans* cells were initially grown at 30°C to an optical density at 600 nm (OD_600_) of 0.5–1.0 in tissue culture flasks containing 7H9 broth supplemented with 0.2% glycerol, 10% OADC and 0.05% (vol/vol) Tyloxapol. Aliquots of 500 μL were then stored at -80°C and the number of colony forming units (CFU) in the freeze stock enumerated. Every experiment was performed starting from a new frozen stock to avoid excessive passage of the original strain. Cells were also routinely passaged on Middlebrook 7H10 agar plates (Difco) supplemented with 10% (vol/vol) OADC to ensure purity of the isolate.

Rifampicin (R3501-1G; Lot Number: SLBH7862V) and meropenem (M2574; Lot Number: 055M4705V) were purchased from Sigma. GlaxoSmithKline provided clarithromycin, streptomycin, clavulanate and all other beta-lactams used in this study.

### Drug susceptibility assays

Minimal Inhibitory Concentrations (MIC) were determined in 7H9 broth supplemented with 0.2% glycerol, 10% OADC and without Tyloxapol using triplicate two-fold serial dilutions of compounds in polystyrene 384- or 96-well plates. MTT [3- (4,5-dimethylthiazol-2-yl)-2,5-diphenyl tetrazolium bromide] was used as the bacterial growth indicator [30, 42]. For cell density calculations, a culture having an OD_600_ of 0.125 was found to contain approximately 10^7^ cfu/mL. Cultures were sampled (50 μL in 384-well plates or 200 μL in 96-well plates) at a final cell density of 10^6^ cfu/mL and incubated at 30°C in the presence of the drug (or drug combinations) for 6 days before addition of 12.5 μL (384-well plates) or 30 μL (96-well plates) of a MTT / Tween 80 (5 mg/mL / 20%) solution mix. After further overnight incubation at 37°C, OD_580_ was measured. The lowest concentration of drug that inhibited 90% of the MTT color conversion (IC_90_) was used to define MIC values.

### Synergy assays

Checkerboard assays and calculations of the Fractional Inhibitory Concentration Index (FICI) were used to define the degree of pairwise drug interactions, as previously described [28] (for a visual representation and deeper understanding of the checkerboard assay refer to the supplementary information of Ramón-García *et al*.[30]). Up to quadruple combinations of rifampicin, clarithromycin, beta-lactams and clavulanate were also tested. For this, checkerboard plates were prepared with rifampicin in the y-axes, the beta-lactam in the x-axes, and clarithromycin added in the z-axes as fixed sub-MIC (1/2, 1/4, and 1/8xMIC values) concentrations for every checkerboard plate; typically the 1/8xMIC plate was used for synergy calculations of the quad combos. When assayed, clavulanate was added at a fixed sub-MIC concentration of 5 μg/mL. Increase efficacy of compounds (synergistic MIC, MIC_syn_) when in combination (fold-MIC reduction) was always reported versus the activity of drugs alone. The Most Optimal Combinatorial Concentration (MOCC) was defined as the lowest possible concentration of every compound that, when assayed together, prevented bacterial growth, i.e. in an isobologram representation this would be the closest point to the axes intersection.

## Results

### Rifampicin has strong synergistic interactions with beta-lactams but not with current anti-BU drugs

**Fig 1:**
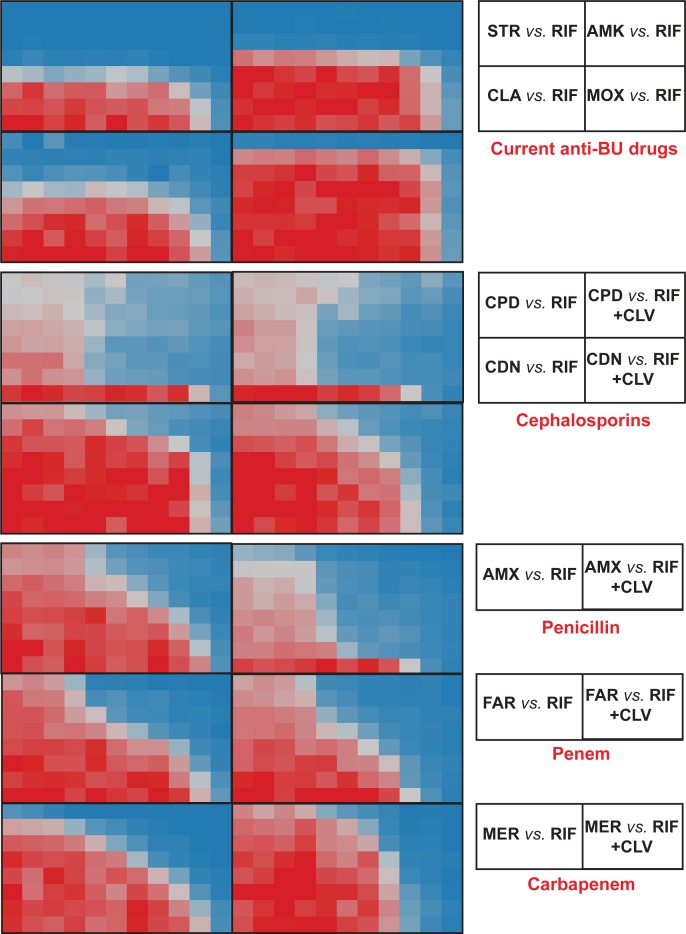
Synergistic profiles of current anti-BU drugs and selected beta-lactams with rifampicin against *M*. *ulcerans* ATCC 19423. Compounds were assayed in two-fold serial dilutions in a checkerboard format. Rifampicin was assayed in the x-axis while other drugs were assayed in the y-axis. RIF, rifampicin; STR, streptomycin; AMK, amikacin; CLA, clarithromycin; MOX, moxifloxacin; CPD, cephradine; CDN, cefdinir; AMX, amoxicillin; FAR, faropenem; MER, meropenem; CLV, clavulanate.

Clinically approved beta-lactams representing different sub-families, i.e. meropenem (carbapenems), cephradine and cefdinir (cephems), faropenem (penems) and amoxicillin (penicillins) were assayed *in vitro* in a checkerboard format to assess their synergistic interactions with rifampicin in the absence and presence of clavulanate, a beta-lactamase inhibitor, against the *M*. *ulcerans* ATCC strain. A pattern of strong synergistic interaction was observed between rifampicin and all beta-lactams tested (**Fig 1**); however, no interaction was observed when the same assay was conducted using combinations of rifampicin and the currently WHO recommended anti-BU drugs, i.e. streptomycin, clarithromycin and moxifloxacin (**Fig 1** and **S1 Fig**). Dose-response curves indicated that the activity of rifampicin (reflected in MIC reduction) was increased on average 16-32-fold (up to 128-fold in some cases) and, vice versa, the activity of the beta-lactams was strongly enhanced by rifampicin. In the case of amoxicillin, its activity was further increased 512-fold in combination with clavulanate (**S2 Fig** and **S1 Table**).

**Fig 2:**
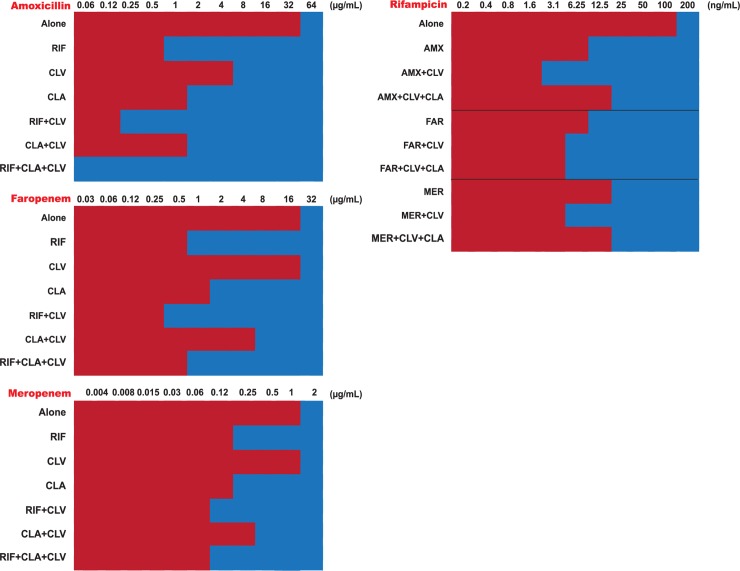
Multiplicative effects of quadruple synergistic combinations including rifampicin, clarithromycin, clavulanate and different beta-lactams against *M*. *ulcerans* ATCC 19423. The MIC of each compound was compared alone and in the presence of several synergistic combinations at the MOCC (lowest FICI). Clarithromycin is not displayed since it was tested at a fixed 1/8xMIC concentration (MIC_CLA_ = 0.5 μg/mL). Clavulanate was tested at a fixed 5 μg/mL concentration. AMX, amoxicillin; CLA, clarithromycin; CLV, clavulanate; FAR, faropenem; MER, meropenem; RIF, rifampicin.

Beta-lactams not only had synergistic interactions with rifampicin but also with clarithromycin, the second drug recommended as first-line anti-BU therapy. These results prompted us to test the inhibitory effect of double clarithromycin-beta lactam, and triple rifampicin-clarithromycin-beta-lactam combinations (**Fig 2**). Our results indicated that, when in double or triple combinations, much lower sub-inhibitory concentrations were equally potent at inhibiting *M*. *ulcerans* growth than the additive effects of the compounds alone. MIC values were also lower than in other pairwise combinations. For example, the MIC of amoxicillin was greater than 32 μg/mL; however, its synergistic MIC (MIC_syn_) was reduced to 1 μg/mL in the presence of rifampicin, to 0.25 μg/mL when clavulanate was also added, or to 0.062 μg/mL when both clavulanate and clarithromycin were included together with rifampicin, i.e., an MIC reduction of ca. 500-fold for amoxicillin when in the quadruple combination. Similar results were obtained for combinations of meropenem or faropenem and rifampicin, with MIC reductions as high as 80-fold when tested within triple combinations. In these assays, clarithromycin was added at a fixed concentration of 1/8 its MIC value alone, being its presence critical to achieve the multiplicative effect observe in the quadruple combinations.

### Amoxicillin/clavulanate is highly active against *M*. *ulcerans* clinical isolates in combination with rifampicin and clarithromycin

Our initial discoveries described above were performed using *M*. *ulcerans* ATCC 19423, a reference strain that does not produce mycolactone, a polyketide-derived macrolide secreted by *M*. *ulcerans* and responsible for the tissue damage pathology in Buruli ulcer [43]. In order to validate the pattern of synergistic interactions between rifampicin, clarithromycin and beta-lactams and to gauge the potential of introducing a beta-lactam in the treatment of BU, we expanded our analysis to a collection of *M*. *ulcerans* clinical isolates from different geographical locations and focused our synergy interaction studies on the amoxicillin /clavulanate combination. When in the quadruple combinations, the activities of all three drugs (clavulanate was added at a fixed 5 μg/mL concentration, more than 20-fold less its MIC) were strongly enhanced; depending on the strain tested, these interactions could range from ca. 5 to 600-fold (rifampicin), ca. 4 to 2,000-fold (amoxicillin) and ca. 20 to 80-fold (clarithromycin) (**Table 1**). Every possible pair-wise and triple combination was also evaluated showing strong synergism between amoxicillin and both rifampicin and clarithromycin, but not between rifampicin and clarithromycin, similar to previously described for the ATCC strain. Clavulanate enhanced the activity of amoxicillin (as expected) but had no effect over clarithromycin and only minor enhancements (in some cases) over rifampicin (**S3 Table**).

**Table 1:** Quadruple synergistic combinations among rifampicin, clarithromycin, amoxicillin and clavulanate against *M*. *ulcerans* clinical isolates.

		MIC (μg/mL) alone			MIC_syn_ (μg/mL) at the MOCC			Fold-Reduction at the MOCC			
Clinical isolate	Geographical origin	RIF	AMX	CLA	RIF	AMX	CLA	RIF	AMX	CLA	Quad FICI
ITM 063846	Benin	0.025	>32	0.125	0.0031	1	0.003	8	64	40	0.20
ITM 070290	China	0.5	>32	1	0.0016	0.063	0.05	313	1024	20	0.09
ITM 83720	Mexico	0.1	16	0.125	0.0063	0.063	0.003	16	256	40	0.13
ITM 941327	Australia	0.002	>32	0.5	0.0004	0.031	0.025	5	2065	20	0.29
ITM C05142	Australia	0.1	16	0.125	0.0016	0.063	0.0031	64	256	40	0.08
ITM C05143	Mexico	0.05	16	0.25	0.0008	4	0.0031	64	4	80	0.32
ITM C05150	DR Congo	0.02	>32	0.25	0.0013	0.063	0.0031	16	1024	80	0.12
ITM C08756	Japan	0.2	>32	0.25	0.0016	0.250	0.0125	128	256	20	0.10
ITM M000932	Australia	0.05	>32	0.063	0.0001	0.125	0.0031	640	256	20	0.09

Clavulanate was added to the quadruple combination at a fixed dose of 5 μg/mL. For FICI calculations, its MIC was considered as 128 μg/mL. RIF, rifampicin; AMX, amoxicillin; CLA, clarithromycin; MOCC, Most Optimal Combinatorial Concentration; Fold-Reduction, MIC/MIC_syn_; FICI ≤0.5 indicates synergy.

**Fig 3:**
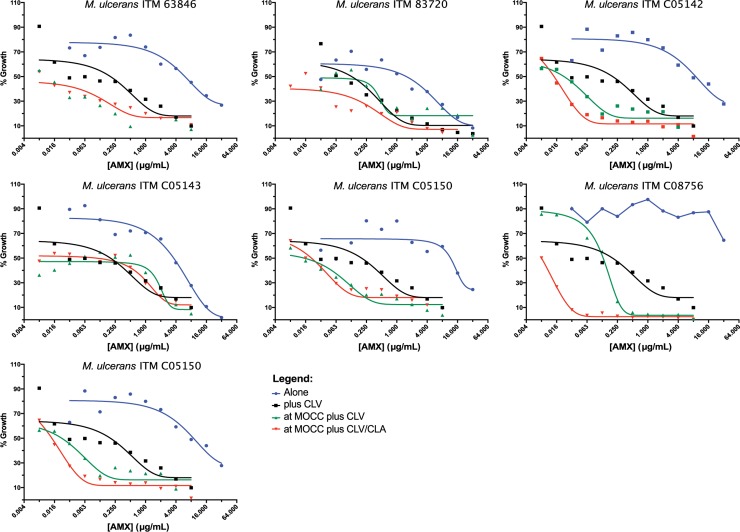
Dose response curves of amoxicillin alone and in combination against *M*. *ulcerans* clinical isolates. Dose response curves of amoxicillin tested: "alone", alone; "Plus CLV", in the presence of clavulanate; "at MOCC plus CLV", in the presence of clavulanate and rifampicin at the MOCC; "at MOCC plus CLV/CLA", in the presence of clavulanate and rifampicin and clarithromycin at the MOCC. Clavulanate was tested at a fixed 5 μg/mL concentration. AMX, amoxicillin; CLA, clarithromycin; CLV, clavulanate.

Amoxicillin is inactivated by beta-lactamase enzymes, limiting its clinical use. In fact, dose-response studies demonstrated that amoxicillin alone was not active (MIC > 16 μg/mL); however, a strong shift in the dose-response curve was observed by adding clavulanate and this shift was further enhanced when clarithromycin and rifampicin were also present in the combination at sub-MIC concentrations (**Fig 3**), thus confirming our MIC/synergy data and the potential of amoxicillin/clavulanate as a new anti-BU therapy, both alone and in combo with rifampicin, clarithromycin or both.

## Discussion

We have *in vitro* characterized the antimicrobial interactions of rifampicin with anti-BU drugs and beta-lactams and found that, while there was no synergy between rifampicin and current anti-BU drugs, it had strong synergistic interactions with beta-lactams. What is more, beta-lactams also displayed synergism with clarithromycin, the other first line drug in BU therapy. Our studies confirmed previous data showing lack of *in vitro* interaction between rifampicin and clarithromycin [44], thus reinforcing synergy data observed with beta-lactams. Similar observations of no interaction between rifampicin and clarithromycin were also recently described in murine models of *M*. *ulcerans* infection [37]

Working with *M*. *ulcerans* is challenging due to its slow generation time (ca. 48 hours), even slower than *M*. *tuberculosis*. Current methodologies to perform susceptibility testing against clinical isolates are often time consuming and cumbersome (use of agar proportion methods), thus, requiring several months to generate results [6, 8, 26]. Improvements have been introduced using luminescent reporter strains [45]; however, this technology is limited to specific engineered strains and cannot be widespread applied to clinical isolates. Here, we were able to perform synergy studies, obtaining results in just seven days, in a medium-throughput manner against a panel of clinical isolates by adapting a methodology previously described for TB research and already validated to determine MIC values in slow growing mycobacteria [28]. Similar redox-based assays (using alamar blue) have been previously employed in antimicrobial discovery programs targeting *M*. *ulcerans* [46, 47]. This methodology could provide a cost and time effective assay to implement in the clinical practice in Buruli ulcer drug resistance surveillance campaigns.

Over the last decade, BU therapy has undergone a tremendous improvement with the introduction of chemotherapy; however, there is only a limited number of drugs recommended by WHO for BU therapy, namely rifampicin, streptomycin, clarithromycin and moxifloxacin [2]. Current WHO recommended therapy is a fully oral eight weeks daily regimen with a combination of rifampicin and clarithromycin [20]. Although effective and mostly well tolerated, combination treatment of rifampicin plus streptomycin or clarithromycin is associated with undesirable side effects that might include mild (anorexia, nausea, abdominal pains and altered taste) or severe (deafness, skin rashes, jaundice, shock, purpura or acute renal failure) symptoms [2]. In a scenario where administration of any of these drugs needs to be interrupted, therapeutic options would remain limited to the use of moxifloxacin, an antibiotic contraindicated during pregnancy and within the pediatric population. A similar situation would occur in the eventual development of resistance to any of these drugs, especially rifampicin. Since they are administered in pairwise combinations, this would effectively imply monotherapy, further promoting the emergence of resistance. This is similar to another mycobacterial disease, leprosy, for which this threat was largely ignored and just recently WHO issued guidelines including procedures for the detection of drug resistance [48]. Thus, an alternative drug regimen would be required to treat resistant *M*. *ulcerans* strains [26], even though the emergence of resistance in *M*. *ulcerans* might follow different dynamics compared to *M*. *tuberculosis* and *M*. *leprae* (environment *vs*. host reservoirs, respectively) and clinical resistance has not been conclusively demonstrated to date.

BU is a neglected disease mainly affecting rural areas in under-resourced countries where medicine access and logistics might prove difficult, and hospitalization and loss of income for patients and families might compromise patient’s adherence to the 8-weeks antibiotic course. A shortened, highly effective, all-oral regimen is urgently needed to improve care for this neglected tropical disease; this would reduce indirect costs and barriers to therapy. The history of TB chemotherapy teaches us that combination therapy is critical for optimal cure outcome and treatment shortening [49]. Translation of this knowledge into BU therapy suggests that more drugs need to be added to the current rifampicin-clarithromycin combination in order to improve and shorten the duration of treatment.

Most beta-lactams tested in this study were active against *M*. *ulcerans* and enhanced the anti-BU activity of rifampicin to different degrees. Cephradine is a first-generation cephalosporins developed in the 1960s, also recently described to be active *in vitro* against *M*. *tuberculosis* [28]; however, cephradine was long ago discontinued and access to other first-generation cephalosporins, such as cefadroxil or cephalexin, is limited in many countries. Cefdinir is a third-generation cephalosporin active against pneumonia, skin and soft tissue infections, although with low oral absorption [50]. It is currently used in the clinic, widely distributed and access to it could be readily available; however, its synergistic profile with rifampicin was weaker compared to other beta-lactams (**S1 Table**). Although meropenem was active against pulmonary TB in a recent clinical study [29], it needs to be administered intravenously, not a practical approach in under-resourced countries where oral drugs are required. Faropenem, an orally administered beta-lactam, did not show activity in the same clinical trial due to the low drug exposure in plasma after oral administration [29]. Finally, amoxicillin/clavulanate showed good activity and very strong synergistic interaction with rifampicin.

The combination of amoxicillin plus clavulanate is a broad-spectrum antibacterial available for clinical use in a wide range of indications and is now used primarily in the treatment of community-acquired respiratory tract infections [51]. It was first launched in the UK in 1981; by the end of 2002, it was clinically available in various formulations in over 150 countries around the world. In addition to high efficacy, it has a well-known safety and tolerance profile, including for pregnancy and pediatric used, based on over 819 million patient courses worldwide, with the main contraindication being allergy to penicillin derivatives. Disruption of the gut microbiota is the main side effect of long-term use of amoxicillin/clavulanate, mainly caused by the presence of clavulanic acid in the formulation [52]. For TB treatment, amoxicillin/clavulanate is included in Group 5 (anti-TB drugs with limited data on efficacy and long-term safety in the treatment of drug-resistant TB) of the WHO 2011 TB drugs classification and in Group D3 (add-on agents, not core MDR-TB regimen components) of the WHO 2016 MDR-TB drugs classification [53]. In 1983, Cynamon *et al*. reported the *in vitro* bactericidal activity of amoxicillin/clavulanate against 15 isolates of *M*. *tuberculosis*, at concentrations of amoxicillin lower than 4 μg/mL [54], and some years later, Nadler *et al*. case reported the effective treatment of MDR-TB patients with the addition of amoxicillin/clavulanate to the second-line therapy [55]. Two contradictory follow up clinical studies, 2-days Early Bactericidal Activity (EBA), suggested that its activity was comparable to that reported for anti-TB agents, other than isoniazid [56], but also questioned its role in the treatment of tuberculosis [57]. The dosing interval of the amoxicillin/clavulanate therapy might explain these differences; while in the first EBA it was divided into three daily doses, it was given as a single high dose in the second one. More recently, a 14-days EBA study demonstrated activity of a combination of meropenem plus amoxicillin/clavulanate [29]; it remains to be determined whether this activity was due to any of the components alone or the combination therapy as a whole [58]. In fact, *in vitro* studies have demonstrated synergistic interactions among amoxicillin and meropenem (and other beta-lactams), rifampicin and ethambutol against *M*. *tuberculosis* [28, 58–60].

In our *in vitro* assays with *M*. *ulcerans* clinical isolates, we found that amoxicillin had no activity (typically MIC values > 16 μg/mL) but that its MIC could be reduced to 1 μg/mL in the presence of clavulanate (**S3 Table**), similar to previously reported to the closely related *M*. *marinum* species and other non-tuberculosis mycobacteria [61]. It also displayed strong synergistic interactions with rifampicin and clarithromycin and, in quadruple combinations, its activity was enhanced up to 2,000-fold in some cases, with average MIC ranges between 0.031 to 0.25 μg/mL (**Table 1**). For infections caused by other bacterial pathogens, susceptibility breakpoints of amoxicillin/clavulanate are established at ≤ 2 μg/mL (or ≤ 4 μg/mL for high-dose formulations) and mean peak plasma concentrations of amoxicillin range from 7.2 to 17 μg/mL, depending on the formulation [52], well above the synergistic MIC values reported in this work. The bacteriological efficacy of penicillins is dependent on the time its free plasma concentration remains above the MIC (time over the MIC value, fT>MIC). For other bacterial infections, it has been estimated that a fT>MIC of ca. 30–40% of the dosing interval is required for bactericidal activity [62]. In the case of *M*. *ulcerans*, this target therapy could be achieved using standard amoxicillin/clavulanate formulations of 500/125 mg (4:1) or 875/125 mg (7:1) administered three times a day, or the high-dose extended release formulation of 2000/125 (16:1) that would allow administration twice a day [52], an important consideration for treatment compliance in under-resourced settings. Thus, according to our *in vitro* data, amoxicillin/clavulanate could have an important role in the treatment of BU alone and, more importantly, in combination with current first-line anti-BU therapy since no pharmacological drug-drug interactions are described among amoxicillin/clavulanate and rifampicin or clarithromycin [51].

But, what could be the benefit of adding amoxicillin/clavulanate to the current anti-BU therapy? Besides being able to treat secondary infections associated with BU lesions, it has been proposed that the median time to healing is related to the bacterial load in the lesions at the beginning of therapy and the presence of persister bacteria [63]; in fact, healing of up to two thirds of patients occurs within 25 weeks from the start of treatment but for some patients this can take up to a year. One of the reasons for this slow healing could be due to a high initial bacterial load. In fact, active infection late into the recommended 8-week course of antibiotic therapy could be found in slowly healing lesions [22, 64]. Extensive histopathologic studies also demonstrated that *M*. *ulcerans* is essentially confined in extracellular areas of necrosis in skin [6]. Under this circumstances, amoxicillin/clavulanate would be extremely effective at targeting extracellular bacteria with rapid bactericidal activity, thus reducing initial bacterial burden, local levels of the immune-suppressive mycolactone toxin, and allowing local recovery of the host immune response to clear remaining bacteria. What is more, *in vitro* studies have demonstrated the sterilizing activity of synergistic combinations of beta-lactams and rifampicin [28], which could target those remaining persistent populations, thus shortening treatment and healing times. Rapid bacterial killing would also imply a reduction in the risk of development of resistance; even in the scenario of infections caused by bacteria resistant to rifampicin, this could still be re-introduced for BU therapy if it was administered with amoxicillin/clavulanate, as previously demonstrated in *M*. *tuberculosis* [28]. Finally, because of their synergistic interactions with clarithromycin, it could replace rifampicin in the treatment of HIV patients under anti-retroviral therapy.

Our study comes as well with some limitations. First, although representative of different geographical origins, we only tested one ATCC strain and 9 clinical isolates. Further *in vitro* studies with a larger set of *M*. *ulcerans* clinical isolates would be needed to assess the full clinical potential and coverage of a triple combination including rifampicin, clarithromycin and amoxicillin/clavulanate. Second, our results were generated using synergy assays based on MIC determinations. This implies two limitations: (i) although an established approach in antimicrobial synergy assays, the activities of drugs alone and in combination were determined at a single time point after seven days of drug exposure and, (ii) synergy calculations inherently rely on sub-MIC concentrations, instead of actual serum levels achieved by drugs in clinical therapy. In order to thoroughly assess the effect of an eventual rifampicin, clarithromycin and amoxicillin/clavulanate combination at therapeutic concentrations, time kill assays would be required. However, even these assays would be challenging; since serum levels would be much higher than MIC values, the individual activity of drugs would mask any synergy signal using bactericidal activity as endpoint readout. Under these circumstances, and the much longer generation time of *M*. *ulcerans* compared to *M*. *tuberculosis*, assessing the sterilization capacity of such combinations would require extensive (months) incubation times of *M*. *ulcerans* cultures. Nevertheless, time kill assays are static pharmacokinetic (PK) / pharmacodynamics (PD) models where drugs are only added at the beginning of the assays and do not reproduce clinical therapy. The hollow fiber system, a dynamic PK/PD model by which posology and length of treatment can be mimicked *in vitro*, might provide data with higher prediction potential of treatment outcomes. This technology has proven a useful tool in the TB field, recently endorsed by the European Medicines Agency [65]; however, to date no laboratory has reported work on *M*. *ulcerans* using the hollow fiber system. Reasons for this might include the difficulties of working with a BSL3 pathogen that forms colonies in 2–4 months. Finally, in the field of BU (and TB), it is common practice to perform preclinical evaluation of drugs (or drug combinations) identified by *in vitro* studies using murine models of *M*. *ulcerans* infection. Although promising, experience from TB research has revealed inconsistencies between murine model data and clinical predictability [66, 67]. In addition, mice are a sub-optimal *in vivo* model to evaluate the activity of beta-lactams since their pharmacokinetics and efficacy in mice do not predict those found in humans [68]; this is in part due to the fact that mice express an enzyme that degrades beta lactams (renal dehydropeptidase I, DPH-I) at levels that are several orders of magnitude higher than in humans [69, 70], thus effectively reducing the time beta-lactams are over the MIC value. As such, murine models might not be the most appropriate development strategy for the use of beta-lactams in BU therapy. Because amoxicillin/clavulanate is a well-known antimicrobial with a clear track record of safety over decades of use, we believe that direct evaluation in clinical trials would be the fastest route to improve treatment of BU patients.

In summary, using a repurposing approach and *in vitro* technology already developed in TB R&D programs, we have identified amoxicillin/clavulanate as a new potential anti-BU drug to be used alone or in combination therapy with rifampicin and clarithromycin, current first-line anti-BU drugs, with the potential to reduce length of therapy and time to healing. Based on the strong synergistic interactions among amoxicillin with rifampicin and clarithromycin, amoxicillin alone might be added to the full course of a shorter therapy. However, because the main role of amoxicillin/clavulanate in the anti-BU therapy would be to reduce the initial bacterial load found in the lesions, we propose the use of high-dose extended release formulations during the first two weeks of therapy.

## Supporting information

S1 Fig Dose response curves of rifampicin in the presence of anti-BU drugs against *M*. *ulcerans* ATCC 19423. (XLSX)

S2 Fig **Dose response curves of rifampicin in the presence of beta-lactams, and vice versa, without (top panel) and with clavulanate (bottom panel)**.
(XLSX)

S1 Table Synergistic interactions among rifampicin and beta-lactams against *M*. *ulcerans* ATCC 19423. (XLSX)

S2 Table Quadruple synergistic combinations among rifampicin, clarithromycin, beta-lactams and clavulanate against *M*. *ulcerans* ATCC 19423. (XLSX)

S3 Table Quadruple synergistic combinations among rifampicin, clarithromycin, beta-lactams and clavulanate against *M*. *ulcerans* clinical isolates. (XLSX)
